# The Supportive Care Needs of Regional and Remote Cancer Caregivers

**DOI:** 10.3390/curroncol28040266

**Published:** 2021-08-09

**Authors:** Anna Stiller, Belinda C. Goodwin, Fiona Crawford-Williams, Sonja March, Michael Ireland, Joanne F. Aitken, Jeff Dunn, Suzanne K. Chambers

**Affiliations:** 1 Viertel Cancer Research Centre, Cancer Council Queensland, Fortitude Valley, Brisbane, QLD 4006, Australia; belindagoodwin@cancerqld.org.au (B.C.G.); fiona.crawfordwilliams@qut.edu.au (F.C.-W.); joanneaitken@cancerqld.org.au (J.F.A.); jeff.dunn@pcfa.org.au (J.D.); 2 Centre for Health Research, University of Southern Queensland, Springfield, QLD 4300, Australia; sonja.march@usq.edu.au (S.M.); michael.ireland@usq.edu.au (M.I.); 3 Cancer Palliative Care Outcomes Centre, Queensland University of Technology, Kelvin Grove, QLD 4059, Australia; 4 School of Psychology and Counselling, University of Southern Queensland, Ipswich, QLD 4305, Australia; 5 School of Public Health and Social Work, Queensland University of Technology, Kelvin Grove, QLD 4059, Australia; 6 School of Public Health, The University of Queensland, Springfield, QLD 4300, Australia; 7 Menzies Health Institute Queensland, Griffith University, Mt Gravatt, QLD 4222, Australia; 8 Prostate Cancer Foundation of Australia, St Leonards, Sydney, NSW 2065, Australia; 9 Faculty of Health, University of Technology Sydney, Ultimo, Sydney, NSW 2007, Australia; suzanne.chambers@acu.edu.au; 10 Faculty of Health Sciences, Australian Catholic University, Banyo, Brisbane, QLD 4014, Australia; 11 Exercise Medicine Research Institute, Edith Cowan University, Joondalup, Perth, WA 6027, Australia

**Keywords:** cancer, caregivers, supportive care needs, rural, regional and remote

## Abstract

Objective: As cancer survival rates continue to increase, so will the demand for care from family and friends, particularly in more isolated settings. This study aims to examine the needs of cancer caregivers in regional and remote Australia. Methods: A total of 239 informal (i.e., non-professional) cancer caregivers (e.g., family/friends) from regional and remote Queensland, Australia, completed the Comprehensive Needs Assessment Tool for Cancer Caregivers (CNAT-C). The frequencies of individuals reporting specific needs were calculated. Logistic regression analyses assessed the association between unmet needs and demographic characteristics and cancer type. Results: The most frequently endorsed needs were lodging near hospital (77%), information about the disease (74%), and tests and treatment (74%). The most frequent unmet needs were treatment near home (37%), help with economic burden (32%), and concerns about the person being cared for (32%). Younger and female caregivers were significantly more likely to report unmet needs overall (OR = 2.12; OR = 0.58), and unmet healthcare staff needs (OR = 0.35; OR = 1.99, respectively). Unmet family and social support needs were also significantly more likely among younger caregivers (OR = 0.35). Caregivers of breast cancer patients (OR = 0.43) and older caregivers (OR = 0.53) were significantly less likely to report unmet health and psychology needs. Proportions of participants reporting needs were largely similar across demographic groups and cancer type with some exceptions. Conclusions: Caregiver health, practical issues associated with travel, and emotional strain are all areas where regional and remote caregivers require more support. Caregivers’ age and gender, time since diagnosis and patient cancer type should be considered when determining the most appropriate supportive care.

## 1. Introduction

Cancer, as a disease group, is the leading cause of disease burden in Australia and the economic cost of cancer to the health system is substantial [1,2]. Caregivers play an indispensable role in maintaining the livelihood and well-being of cancer patients. A caregiver is defined here as the person (e.g., partner, family member or friend) “nominated by the cancer patient as most involved in supporting them through the illness” [3]. Caregivers help the patient in a myriad of ways such as in managing symptoms and side-effects, providing assistance at medical appointments, giving emotional support, assisting in activities of daily living, as well as taking on extra family and household responsibilities [4].

Caring for someone with cancer, particularly a loved-one, can incur significant physical and emotional burdens for the caregiver [5,6,7]. While concurrently dealing with their own emotional and existential concerns, caregiving demands may at times exceed the caregiver’s capabilities and resources [8]. As cancer survival rates continue to increase, so will the demand for care and support from family and friends. Therefore, it is vital that caregivers’ needs are assessed and addressed in order to support their own well-being and maximize their capacity to provide care for the patient [9].

Not all needs are of equal importance to all caregivers [10]. Age (being younger) and gender (being female) have been associated with a higher frequency of unmet needs among caregivers [3,9,10,11,12]. Kim and colleagues [10] found that younger caregivers were more likely to report unmet psychosocial and financial needs at two years post-diagnosis, and unmet needs for managing loss if they were bereaved caregivers at five years. The patient’s cancer type is also associated with caregiver needs. For example, research has found that caregivers of patients with haematological cancer have more unmet needs compared to caregivers of patients with solid tumours [13]; and caregivers of those with lung cancer and non-Hodgkins lymphoma have a higher frequency of health care service needs and information needs, respectively, compared to other cancer types [3].

There are unique burdens and challenges for caregivers living in regional and remote areas [14,15,16]. They may experience poorer access to health care and support services, difficulties coping with the logistical challenges of regular travel or relocation during the patient’s treatment, disruption to work capacity, financial instability, and social disconnection [14,15,16]. Furthermore, in regional and remote communities where access to medical facilities is limited, caregivers may be required to take on roles that in urban areas would be fulfilled by professional support services. Rural and remote areas typically lack the infrastructure, specialist and allied health services found in more densely populated urban areas. As a result the increase in early discharge procedures in the healthcare system may have a considerable impact on caregivers in rural and remote areas, who are taking up the majority of the day-to-day responsibility during the patient recovery and rehabilitation period [17,18]. Consequently, the magnitude of caregiver needs may also vary depending on geographical location. For instance, among a sample of caregivers of people with haematological cancers, those living in rural areas had a higher prevalence of finance-related unmet needs compared to those from urban areas (e.g., unmet need in ‘finding information about financial help’—rural caregivers 20% vs. urban 13% [15]). Therefore, it is essential to establish the importance of varying needs in this group and investigate any difference according to demographic, geographical, and disease characteristics. In order to tailor appropriate support and information resources to cancer caregivers from regional or remote areas, identifying and addressing the support needs of cancer caregivers in regional and remote communities is particularly important and a vital element in improving health and well-being outcomes in these areas.

The aim of this study is to describe the types and prevalence of supportive care needs of cancer caregivers in regional and remote Australia, both in terms of prevalence of needs and degree of unmet need and to compare unmet need according to the caregiver’s age group, gender, geographical location, socio-economic status, the length of time since diagnosis, and the cancer type of the patient. This information will assist in identifying areas where support is most needed for cancer caregivers living in regional and remote communities and may help to inform future supportive care interventions.

## 2. Materials and Methods

### 2.1. Participants

Participants were the nominated caregivers of cancer patients recruited to a larger research project. The cancer patients were staying at subsidised accommodation lodges while receiving their cancer care in a major centre more than 50 km from their home. Details of patient recruitment is detailed elsewhere [19]. Caregivers included spouses/partners, family members, or friends. The eligibility criteria included being over 18 and being able to read and understand English. Caregivers did not have to accompany the cancer patient to the accommodation lodge, and did not have to be living in the same personal residence as the cancer patient, in order to be recruited to the study. They were not professional paid caregivers; however, it is possible that some may have been claiming a carer’s payment from the government (this information was not collected).

### 2.2. Recruitment

**Figure 1:**
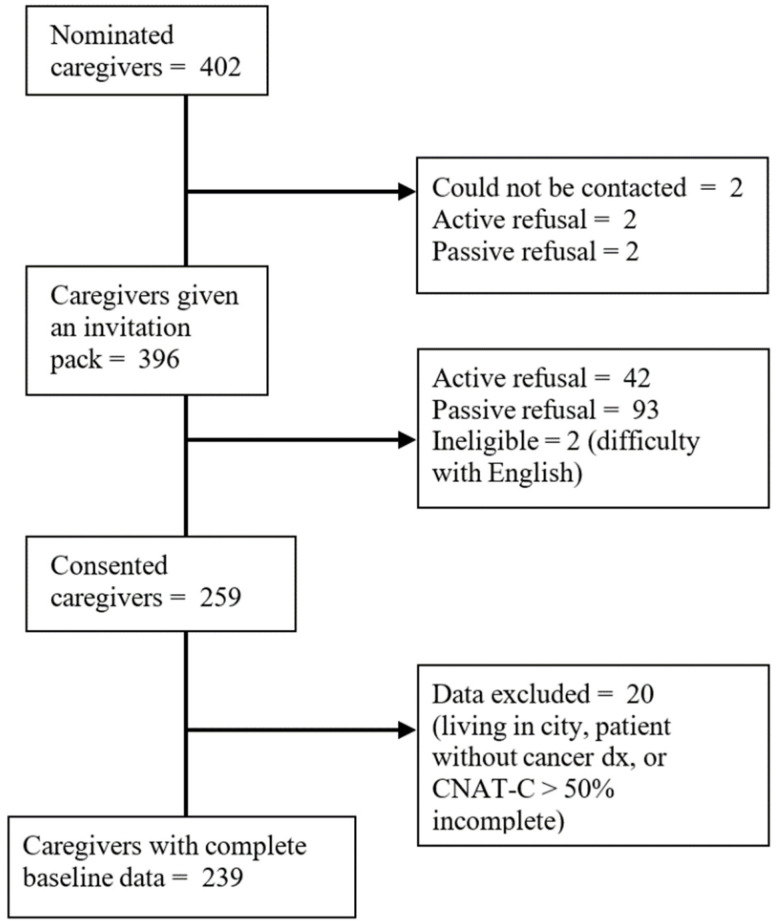
Recruitment flowchart.

Cancer patients participating in the larger study nominated 402 caregivers and provided their contact details. The research team contacted nominated caregivers via telephone, inviting them to participate and sending out an invitation pack, which included consent forms and a questionnaire, if they were interested in participating. Of these, 259 consented to participate and 239 caregivers provided complete data for the current study. Figure 1 depicts the participant recruitment flow chart.

### 2.3. Materials

Assessments included a self-administered questionnaire (SAQ) and face-to-face or telephone interview at baseline. These instruments captured data such as patient and caregivers’ experiences, psycho-social wellness, and satisfaction with their health care, along with the demographic and caregiver supportive care needs measure used in the current study.

### 2.4. Measures

#### 2.4.1. Caregivers’ Demographic Characteristics

Gender, age, country of birth, and highest level of education were reported by each participant. Their residential street address at baseline was geocoded and mapped to the 2011 SA2 boundaries using MapMarker Australia V.15.16.0.21 and MapInfo Pro V.5.0 and classified by Remoteness Area [20] and Socioeconomic Index for Areas (SEIFA) [21].

#### 2.4.2. Patient Diagnosis Information

The most recently diagnosed primary cancer site of the patient for whom they were caring was obtained via self-report from the patient and verified in the population-based Queensland Cancer Register (QCR). Patient self-report data were relied on where diagnosis could not be verified by the QCR (*n* = 22), for example, if the patient had non-melanoma skin cancer (which is not routinely notified to registries in Australia) or the patient’s diagnosis was very recent and had not yet been notified to the QCR.

#### 2.4.3. Caregiver Supportive Care Needs

There are several useful measures of cancer caregiver need that have been utilised and validated in Australian samples [8,22,23]. However, given the unique challenges for caregivers in regional and remote areas, the Comprehensive Needs Assessment Tool for Cancer Caregivers (CNAT-C) [24] was used in the current study due to the inclusion of a ‘practical support’ domain that covers needs such as treatment near home and transport service, which may be particularly relevant for those living in more remote, underserviced areas. The CNAT-C is a comprehensive needs assessment tool for caregivers of cancer patients, that includes 41 items across 7 domains: health and psychological problems (e.g., need for help with ‘my own health problems’); family/social support (e.g., ‘help with difficulties in family relationships after cancer diagnosis’); health-care staff (e.g., ‘being respected and treated as a person by my doctor’); information (e.g., ‘information about tests and treatment’); religious/spiritual support (e.g., ‘help in finding the meaning of my situation and coming to terms with it’); hospital facilities and services (e.g., ‘need for space reserved for caregivers’); and practical support (e.g., ‘lodging near hospital’). Items were answered on a 5-point Likert scale: “no need/not applicable”; “no need/satisfied”; “low need”; “moderate need”; and “high need”, referring to the past month. The original CNAT-C uses a 4-point scale, however, in order to distinguish those with no need and those who had no need because the need was satisfied, an extra response item was added. (i.e., need satisfied).

Given the CNAT-C has not been validated for use outside of Korea and China [24,25], a confirmatory factor analysis (CFA) was conducted to assess whether the proposed domains were applicable in a regional and remote Australian setting. The CFA, conducted in Mplus v8, used a weighted least-squares mean and variance adjusted (WLMSV) estimator [26]. The CFA demonstrated reasonable fit for the original scale in this sample (χ^2^ = 1545.88, *p* < 0.001, RMSEA = 0.066, *p* < 0.001, CFI = 0.915, TLI = 0.908). All but one item loaded above 0.65 on their respective factors (Table A1 and Table A2). The religious/spiritual support factor contained only two items and the item ‘religious support’ had a factor loading of 0.12 while the second item ‘help in finding the meaning of my situation and coming to terms with it’ had a factor loading of 3.69. Modification indices suggested substantial improvements in model fit could be achieved by removing both of these items, which also demonstrated poor variance and insufficient endorsement in this sample (i.e., 1.67% and 7.11% non-zero responses, respectively). A second model was assessed excluding the religious/spiritual support factor (and the items within). The second model demonstrated better fit (χ^2^ = 1090.81, *p* < 0.001, RMSEA = 0.050, *p* = 0.542, CFI = 0.955, TLI = 0.951) and the factor structure remained stable with all factor loadings above 0.65. Religiosity items were therefore not included in the main analysis in this study.

Multiple scoring approaches were applied to create three versions of the subscale scores. These included a variable reflecting ‘need’ (whether met or unmet), which was calculated whereby 0 = not applicable; and 1 = satisfied, low need, moderate need, or high need. A second variable reflecting ‘Unmet need’ was calculated where 0 = not applicable, or satisfied; and 1 = low, moderate, or high need and a third reflecting ‘Degree of unmet need’ was also calculated for each item where 0= not applicable or satisfied; 1 = low need; 2 = moderate need; and 3 = high need.

### 2.5. Data Analysis

Frequencies and proportions of need (i.e., prevalence of need regardless of being met or unmet) and unmet need for each item, and unmet need for each domain, were calculated. Comparisons of prevalence of unmet need in each domain were made across age, gender, area level and cancer type groupings using logistic regression. In models where age of caregiver was an independent variable, age of patient was controlled for. Where *p* values less than 0.05 indicated significant group differences existed for variables with more than two categories (i.e., age and time since diagnosis), polynomial contrasts were assessed. In addition, frequencies of unmet need for single items were compared through ranking the percentage of participants reporting at least some need for each item across age, gender, area level and cancer type group; a descriptive method applied in previous similar studies for identifying levels of need across groups [5,15,27].

## 3. Results

### 3.1. Sample Characteristics

Most caregivers in the sample were the spouse or partner of the person with cancer (83%), female (62%) and born in Australia (81%). The mean age of caregivers was 62.0 years (standard deviation (SD) = 12.6, range 18–91). The median time since the patient’s diagnosis was 8.9 months (interquartile range: 4.8 to 23.3). Ninety-five percent of the sample were within 84 months (7 years) from the patient’s diagnosis, with one patient being diagnosed with their current primary cancer 25 years ago. Caregivers were mostly caring for patients who had been diagnosed with breast (18%), skin (14%), prostate (12%) or head and neck (11%) cancer. Most resided in inner (52%) and outer (43%) regional areas, with only 5% residing in remote or very remote areas. Caregivers resided in predominantly low socio-economic areas. See Table 1 for further details of the demographic characteristics of caregivers in this sample.

### 3.2. Overall Caregiver Needs

Overall, 96.2% of caregivers reported that a need existed for them (regardless of whether it had been met) in at least one domain over the past month, with 71.6% reporting at least one unmet need over the past month. The majority of caregivers reported that at least one need existed for them in the practical support (86.6%), information (86.2%), health-care staff (82.4%) (e.g., ‘seeing doctor quickly and easily when in need’), health and psychology (80.8%), and hospital facilities and services (74.5%) domains (see Table 2). Unmet need was most frequently reported in the practical support (50.2%), health and psychological (48.5%), and information domains (45.6%), whereas healthcare staff (30.5%), family/social support (32.6%), and hospital facilities and services (35.2%) were domains where need was less often reported as unmet (see Table 3).

In terms of single items, the most frequently endorsed needs were lodging near hospital (76.7%); information about tests and treatment (74.0%); information about the current status of the illness (73.8%); and concerns about the person being cared for (71.2%; see Table 2). The most frequently reported unmet needs were treatment near home (36.8%), help with economic burden caused by cancer (31.8%), and concerns about the person being cared for (31.8%). Mean degree of unmet need for each item ranged from 0.12 to 0.85, with treatment near home (M = 0.85, SD = 1.22), help with economic burden (M = 0.62, SD = 1.03), information about financial support for medical expenses (M = 0.59, SD = 1.02), lodging near hospital (M = 0.57, SD = 1.08), and concerns about the person being cared for (M = 0.54, SD = 0.91) being the items with the highest mean degree of unmet need.

### 3.3. Comparing Overall and Domains of Unmet Need

Logistic regression analyses suggested that overall unmet need was more frequently reported by female (OR = 2.12, 95% CI = 1.18, 3.79) and younger (OR = 0.58, 95% CI = 0.34, 0.99) caregivers. Practical support was the most commonly reported domain of need for all but a few sub-groups (see Table 3). Unmet health and psychology needs were significantly less likely to be reported by caregivers of breast cancer patients (OR = 0.43, 95% CI = 0.20, 0.94) and older caregivers (OR = 0.53, 95% CI = 0.28, 1.00). Unmet healthcare staff needs were more frequently reported by younger (OR = 0.35, 95% CI = 0.17, 0.70) and female caregivers (OR = 1.99, 95% CI = 1.09, 3.79). Unmet family and social support needs were more frequently reported by younger caregivers (OR = 0.35, 95% CI = 0.17, 0.71).

### 3.4. Comparing Single Item Unmet Needs

Unmet needs related to ‘treatment near home’ and having unaddressed ‘concerns about the person being cared for’ were consistently reported by at least 20% of participants within each demographic and cancer type group (Table 4 and Table 5). Overall proportions of unmet needs were relatively stable across demographic and cancer type groups, however, there were some noteworthy exceptions.

Caregivers based in outer regional and remote areas most frequently reported an un-met need for ‘treatment near home’ (41.8%), ‘help with economic burden’ (33.0%), ‘information about financial support’ (30.4%), ‘concerns about the person I provide care for (29.7%), and lodging near hospital (25.0%). Caregivers from inner regional areas had a similar pattern, and in addition, at least a quarter of caregivers from inner regional areas reported an unmet need for help with ‘my own health problems’ (32.3%), and ‘help with my own relaxation and my personal life’ (25.0%).

For females, ‘information about financial support’ (32.4%), ‘my own health problems’ (31.0%), ‘lodging near hospital where the person I am caring for is treated’ (27.3%), and ‘feelings of vague anxiety’ (26.2%) were reported as unmet by over a quarter of the sample, whereas less than a quarter of males reported these items.

Among caregivers below the age of 68, at least a quarter reported an unmet need for ‘help with economic burden’, ‘information about financial support for medical expenses’, ‘lodging near hospital’, ‘concerns about the person with cancer’, and ‘feelings of vague anxiety’, whereas 20% or less of the oldest group (>68 years) reported these items as unmet. For the youngest caregiver group (≤57 years), at least a quarter reported ‘help with my own relaxation and my personal life’ (32.4%), ‘transportation service for getting to and from the hospital’ (27.4%), ‘someone to help with housekeeping and/or childcare’ (27.4%), and ‘help with difficulties in family relationships’ (25.7%), but these were less frequent among the older age groups (for specific details refer to Table 4). The most common unmet need for caregivers aged over 68 years was help with ‘my own health problems’ (34.7%), whereas this was less frequent in younger caregivers (26.0% of caregivers ≤57 years of age; 24.7% ≥58 and ≤68 years).

In terms of the time since diagnosis, ‘help with my own relaxation and my personal life’ was reported by over a quarter of caregivers of patients who were diagnosed over six months ago, but was less common within the first six months since diagnosis. In addition, among caregivers of patients diagnosed over twelve months ago, ‘transportation service for getting to and from the hospital’ was reported by 27.2%. Help with ‘my own health problems’ was reported by less than 20% of caregivers of patients with breast cancer and skin cancer, even though it was reported by at least a quarter of caregivers of patients with other cancer types (frequencies ranged between 28.0% and 35.7%, for details refer to Table 5). ‘Help with economic burden caused by cancer’ was reported by 10.7% of caregivers of patients with prostate cancer, whereas it was reported by more than a quarter of caregivers of patients with the other cancer types. Similarly, ‘information about financial support’ was reported as unmet by more than a quarter of most caregivers, except for those caring for patients with gynaecological or prostate cancer. In general, ‘feelings of vague anxiety’, ‘help with my own relaxation and personal life’, and ‘information about caregiving-related stress management’ were reported as unmet by less than a quarter of caregivers. However, among those caring for a patient with ‘other’ cancer types (consisting of the least common cancers in this sample), these items were endorsed by at least 25%. These frequencies still held even after removing colorectal cancer, non-Hodgkins lymphoma, and lung cancer from the ‘other’ cancer group, since they are relatively common cancers although not well-represented in this sample. The same three items representing psychological or emotional distress were endorsed by at least 28% of caregivers of the remaining ‘other’ cancer types (such as brain, oesophageal, stomach, bladder cancer). ‘Feelings of anger, irritability or nervousness’, ‘loneliness or feelings of isolation’, and ‘depression’ were reported by more than a quarter of caregivers of patients with head and neck cancer, yet was less frequent when considering the sample overall.

## 4. Discussion

The current findings provide valuable insight into the areas where caregivers caring for cancer patients in regional and remote Australia require support. Needs that were associated with treatment, staff and facilities were generally well-addressed in this regional and remote sample, whereas a large degree of unmet need was evident in areas relating to practical and psychological support. These findings are consistent with previous studies on patient [27,28] and caregiver [29,30] cohorts that suggest higher levels of satisfaction with health care and facilities during treatment, but poorer access to peripheral support. These findings support calls for better provision of psychosocial and practical support following treatment when regional and remote patients and caregivers return to isolated settings [31]. Unsurprisingly, accommodation near treatment was an important, but well-addressed, need in this cohort of participants recruited through subsided lodges available for the purpose of providing accommodation close to treatment.

Gender differences in unmet need were evident, with female caregivers reporting higher levels of unmet need than males particularly when it came to interactions with healthcare staff. Females were almost two times more likely to report one or more needs in this domain. Previous research has frequently found female caregivers to report greater unmet needs than males [9,11,12,32,33]. This may be interconnected with the greater emotional distress and adverse outcomes reported by female caregivers compared to males, which is another consistent finding in the literature [32,34,35,36]. Although the mechanisms underlying the gender difference are not well understood, theoretical explanations include the impact of internalized traditional gender roles on psychological processes and behaviour [34]. For example, men may be less likely to perceive or disclose their needs for help, compared to women [37]. In addition, men may tend to perceive the caring role as a competency task leading to a positive appraisal and self-mastery and esteem response [32,38], whereas women may tend to hold high internal expectations and a sense of moral obligation related to their caring role, leading to over-responsibility, dysfunctional self-sacrifice, and an absence of self-care [32,33,38,39]. These theories require further evaluation.

Overall, our findings suggested that younger caregivers are slightly more likely to experience unmet needs; however, in terms of specific needs, older caregivers more often experienced unmet needs relating to maintaining their own health. Conversely, younger caregivers were slightly to moderately more likely to report a need for psychosocial support. This may reflect the tendency for people to report slightly better mental health related quality of life and slightly poorer physical quality of life as they age [40]. Furthermore, younger caregivers may be more likely to be employed and have other social roles and family responsibilities, which could lead to them experiencing a heavier load from additional caregiving responsibilities. They may have comparatively less experience with serious illnesses and navigating the health system, and be less likely to consider caregiving a normative task for their age-group. These factors may thus contribute to younger caregivers experiencing greater psychological distress than older caregivers [41].

Descriptive differences in frequencies of unmet need for single items according to cancer type suggest a trend that caregivers of patients with head and neck cancer and other less common cancers who live in regional or remote areas, may benefit from additional psychosocial support. Previous research indicates the psychological and emotional needs of caregivers of head and neck patients are consistently high over time, possibly related to a lack of support, and the complex patient care needs and functional difficulties post-treatment [42]. Similarly, there is evidence that patients with rare cancers may have poorer psychosocial outcomes than the general cancer population [43], which could impact caregivers, however, findings around this are mixed [44]. There is limited empirical research into the psychosocial well-being and needs of caregivers of patients with rare cancers in general [45], highlighting a need for future research on the topic.

### 4.1. Recommendations

The current findings suggest that regional and remote cancer caregivers need extra support to cope, not only with practical issues such as travel, financial losses and household upkeep, but also their mental well-being and concerns regarding the patient they care for. Although providing treatment and counselling facilities closer to home may not be a viable strategy, tele-health and community-based services hold promise in partly addressing these needs [31,46].

Some caregiver and patient characteristics may be important to consider in planning such interventions. That is, tailoring supportive care to target individual and situational characteristics is likely to be as important for caregivers as it is when addressing patient support needs [47,48]. According to the current findings, this may include providing younger caregivers with more information about financial support, while assisting older caregivers with managing their own health. The unique experiences of younger female caregivers should be considered in caregiver-health professional interactions to ensure needs are met in terms of respect and quality communication regarding the patient. Finally, providing caregivers with an opportunity to rest and recover is essential for maintaining their capacity to care for their loved ones [49]. According to the current findings, the respite and emotional needs of caregivers appear to be particularly important in the period 6 to 12 months after diagnosis.

### 4.2. Strengths and Limitations

This study utilised a relatively large sample of caregivers when compared to similar studies and represents a range of demographic groups within regional and remote settings. It is the first study to apply the multi-domain CNAT-C scale in a Western sample and largely demonstrates validity of the factor structure for use in this setting. Finally, caregivers were supporting a very diverse range of cancer patients, including a variety of cancer types and treatment statuses. This enhances the diversity of the sample however future research may benefit from focusing on specific groups. There are some limitations worth noting. Selection bias is important to consider due to the recruitment process, since it required both cancer patients’ nomination, and caregivers to agree to participate. Furthermore, given that these cancer patients were already utilizing the subsidized accommodation lodges, it is possible that their healthcare access and supportive care needs may be better fulfilled in comparison to the general remote cancer population.

## 5. Conclusions

Maintaining caregiver health, practical issues associated with travel, and emotional strain are all areas where regional and remote caregivers require more support. The age and gender of caregivers, along with time since diagnosis, and cancer type of the patient should be considered in determining the most appropriate supportive care for cancer caregivers.

## Figures and Tables

**Table 1:** Sample characteristics.

	*n* ^1^	%
Gender		
Female	146	61.6%
Male	91	38.4%
Relationship to patient		
Spouse/Partner	164	83.3%
Other relative	23	11.7%
Other non-relative	10	5.1%
Highest level of education		
Year 10 or below	95	42.4%
Senior high school	30	13.4%
Tertiary (Tafe/Uni)	99	44.2%
ATSI		
No	224	98.3%
Yes	4	1.8%
Country of birth		
Australia	165	81.3%
United Kingdom	17	8.4%
New Zealand	10	4.9%
Other	11	5.4%
Area-level disadvantage (SEIFA)		
1st Quintile (lowest)	94	39.5%
2nd	74	31.1%
3rd	45	18.9%
4th	23	9.7%
5th Quintile (highest)	2	0.8%
Remoteness		
Inner regional	124	52.3%
Outer regional	101	42.6%
Remote and very remote	12	5.1%
Cancer type of patient		
Breast	44	18.4%
Skin	34	14.2%
Prostate	28	11.7%
Head and neck	25	10.5%
Gynaecological	21	8.8%
Colorectal	16	6.7%
Lung	11	4.6%
Non-Hodgkins lymphoma	13	5.4%
Brain	5	2.1%
Other	34	14.2%
Unknown	8	3.3%

Abbreviations: ATSI = Aboriginal or Torres Strait Islander; Tafe =Technical and further education institution; Uni = University. ^1^ Total *n* does not always equal 239 where data was missing.

**Table 2:** Frequency of responses to CNAT-C items.

Domain	CNAT-C Item (Ordered by Item Number in Scale)	Need (Including Met) N (%)	Unmet Need N (%)	Degree of Need Mean (SD)
Health and Psych		193 (80.8%)	116 (48.5%)	0.36 (0.59)
	My own health problems	116 (49.2%)	66 (28.0%)	0.43 (0.79)
	Concerns about the person I provide care for	168 (71.2%)	75 (31.8%)	0.54 (0.91)
	Depression	88 (37.5%)	43 (18.3%)	0.28 (0.67)
	Feelings of anger, irritability, or nervousness	102 (43.4%)	47 (20.0%)	0.30 (0.68)
	Loneliness or feelings of isolation	83 (35.2%)	43 (18.2%)	0.28 (0.68)
	Feelings of vague anxiety	120 (50.9%)	56 (23.7%)	0.35 (0.71)
Family/Social		151 (63.2%)	78 (32.6%)	0.25 (0.50)
	Help with over-dependence from the person I am caring for	82 (34.5%)	26 (10.9%)	0.18 (0.55)
	Help with lack of appreciation of my caregiving from the person …	86 (36.3%)	33 (13.9%)	0.23 (0.64)
	Help with difficulties in family relationships after cancer diagnosis	106 (44.4%)	37 (15.5%)	0.24 (0.62)
	Help with difficulties in interpersonal relationships after cancer …	106 (44.4%)	36 (15.1%)	0.21 (0.57)
	Help with my own relaxation and my personal life	126 (52.7%)	54 (22.6%)	0.37 (0.77)
Healthcare staff		197 (82.4%)	73 (30.5%)	0.19 (0.42)
	Being respected and treated as a person by my doctor	105 (43.9%)	18 (7.5%)	0.12 (0.47)
	Doctor to be clear, specific and honest in his/her explanation	132 (55.2%)	28 (11.7%)	0.22 (0.67)
	Seeing doctor quickly and easily when in need	149 (62.6%)	39 (16.4%)	0.30 (0.76)
	Being involved in the decision-making process in choosing any tests or…	143 (59.8%)	27 (11.3%)	0.19 (0.60)
	Cooperation and communication among health care staff	158 (66.1%)	34 (14.2%)	0.26 (0.69)
	Sincere interest and empathy from the nurses looking after the person …	153 (64.0%)	17 (7.1%)	0.13 (0.51)
	Nurses to explain treatment or care that is being given to the person …	158 (66.1%)	20 (8.4%)	0.13 (0.45)
	Nurses to promptly attend to the discomfort and pain of the person…	151 (63.5%)	25 (10.5%)	0.17 (0.54)
Information		206 (86.2%)	109 (45.6%)	0.33 (0.53)
	Information about the current status of the illness of the person I am…	175 (73.8%)	43 (18.1%)	0.37 (0.84)
	Information about tests and treatment	176 (74.0%)	45 (18.9%)	0.36 (0.80)
	Information about caring for the person with cancer …	167 (69.9%)	41 (17.2%)	0.31 (0.74)
	Guidelines or information about complementary and alternative medicine	115 (48.5%)	33 (13.9%)	0.27 (0.74)
	Information about hospitals or clinics and physicians who treat cancer	156 (65.8%)	37 (15.6%)	0.24 (0.62)
	Information about financial support for medical expenses…	165 (69.6%)	70 (29.5%)	0.59 (1.02)
	Help with communication with the person I am caring for and/or …	116 (49.0%)	25 (10.6%)	0.15 (0.48)
	Information about caregiving-related stress management	121 (51.1%)	46 (19.4%)	0.31 (0.71)
Hospital facilities		178 (74.5%)	84 (35.2%)	0.28 (0.54)
	A designated hospital staff member who would be able to provide …	128 (54.2%)	46 (19.5%)	0.37 (0.84)
	Guidance about hospital facilities and services	150 (63.6%)	36 (15.3%)	0.25 (0.67)
	Need for space reserved for caregivers	116 (49.2%)	40 (17.0%)	0.32 (0.79)
	A visiting nurse service for the home of the person I am caring for	61 (25.6%)	27 (11.3%)	0.21 (0.68)
	Opportunity to share experiences or information with other caregivers	84 (35.6%)	31 (13.1%)	0.20 (0.57)
	Welfare services (e.g., psychological counselling) for caregivers	98 (41.5%)	48 (20.3%)	0.33 (0.73)
Practical Support		207 (86.6%)	120 (50.2%)	0.53 (0.73)
	Transportation service for getting to and from the hospital	146 (61.9%)	51 (21.6%)	0.46 (0.97)
	Treatment near home	157 (67.1%)	86 (36.8%)	0.85 (1.22)
	Lodging near hospital where the person I am caring for is treated	181 (76.7%)	57 (24.2%)	0.57 (1.08)
	Help with economic burden caused by cancer	138 (58.5%)	75 (31.8%)	0.62 (1.03)
	Someone to help me with housekeeping and/or childcare	83 (34.9%)	41 (17.2%)	0.34 (0.83)
	Assisted care in hospital or at the home of the person I am caring for	71 (29.8%)	31 (13.0%)	0.24 (0.71)
All above items (excludes religious/spiritual domain)		230 (96.2%)	171 (71.6%)	0.33 (0.44)

… = item truncated.

**Table 3:** Frequency of caregiver unmet need in CNAT-C domains according to patient cancer type and caregiver demographics.

CNAT-C Domain	Overall Sample	Gender		Age			SES		Remoteness		Time since Diagnosis			Cancer Type					
	% (*n*)	Male % (*n*)	Female % (*n*)	≤57 Years % (*n*)	≥58 & ≤68 Years % (*n*)	>68 Years % (*n*)	<50 Percentile % (*n*)	≥50 Percentile % (*n*)	Inner Regional % (*n*)	Outer regional/Remote % (*n*)	0–6 Months % (*n*)	6–12 Months % (*n*)	>12 Months % (*n*)	Breast % (*n*)	Skin % (*n*)	Head & Neck % (*n*)	Prostate % (*n*)	Gynae % (*n*)	Other % (*n*)
Practical support	50.2% (120)	46.2% (42)	52.1% (76)	56.8% (42)	48.2% (41)	44.7% (34)	50.3% (99)	51.2% (21)	52.4% (65)	47.8% (54)	49.4% (42)	48.3% (28)	51.1% (47)	43.2% (19)	52.9% (18)	48.0% (12)	50.0% (14)	52.4% (11)	50.7% (39)
Health and psychological	48.5% (116)	41.8% (38)	52.1% (76)	56.8% (42)	49.4% (42)	40.8% (31)	49.8% (98)	43.9% (18)	50.8% (63)	46.9% (53)	49.4% (42)	41.4% (24)	51.1% (47)	34.1% (15)	41.2% (14)	56.0% (14)	50.0% (14)	42.9% (9)	55.8% (43)
Information	45.6% (109)	40.7% (37)	48.0% (70)	51.4% (38)	48.2% (41)	36.8% (28)	48.2% (95)	34.2% (14)	48.4% (60)	42.5% (48)	49.4% (42)	44.8% (26)	40.2% (37)	40.9% (18)	44.1% (15)	48.0% (12)	42.9% (12)	38.1% (8)	46.8% (36)
Hospital facilities and services	35.2% (84)	28.6% (26)	39.0% (57)	37.8% (28)	37.7% (32)	29.0% (22)	36.0% (71)	31.7% (13)	36.3% (45)	33.6% (38)	35.3% (30)	31.0% (18)	34.8% (32)	27.3% (12)	35.3% (12)	36.0% (9)	28.6% (8)	42.9% (9)	35.1% (27)
Family/social support	32.6% (78)	27.5% (25)	34.9% (51)	47.3% (35)	31.8% (27)	19.7% (15)	35.0% (69)	22.0% (9)	35.5% (44)	30.1% (34)	27.1% (23)	34.5% (20)	34.8% (32)	27.3% (12)	20.6% (7)	28.0% (7)	25.0% (7)	23.8% (5)	42.9% (33)
Health-care staff	30.5% (73)	22.0% (20)	36.3% (53)	39.2% (29)	32.9% (28)	19.7% (15)	31.5% (62)	26.8% (11)	30.7% (38)	31.0% (35)	23.5% (20)	32.8% (19)	33.7% (31)	22.7% (10)	41.2% (14)	24.0% (6)	25.0% (7)	14.3% (3)	36.4% (28)
All domains—any unmet need	71.6% (171)	61.5% (56)	77.4% (113)	78.4% (58)	70.6% (60)	65.8% (50)	73.1% (144)	65.9% (27)	71.8% (89)	71.7% (81)	72.9% (62)	72.4% (42)	68.5% (63)	63.6% (28)	76.5% (26)	72.0% (18)	71.4% (20)	61.9% (13)	74.0% (57)

Abbreviation: Gynae = Gynaecological.

**Table 4:** Frequency of unmet need **^1^** by age, gender, and area level characteristics.

	Overall Sample	Gender		Age			Remoteness		Area-Level Disadvantage (SEIFA)	
CNAT-C Item (25 Most Frequent Overall)	% (Rank)	Male % (Rank)	Female % (Rank)	≤57 Years % (Rank)	≥58 & ≤68 Years % (Rank)	>68 Years % (Rank)	Inner Regional (*n* = 124) %(Rank)	Outer Regional & Remote (*n* = 113) % (Rank)	<50 Percentile (*n* = 197) % (Rank)	≥50 Percentile (*n* = 41) % (Rank)
Treatment near home	36.8% (1)	29.2% (3)	41.3% (1)	41.7% (3)	39.3% (1)	27.0% (2)	32.0% (3)	41.8% (1)	36.3% (1)	40.0% (1)
Help with economic burden caused by cancer	31.8% (2)	34.4% (1)	29.9% (4)	43.8% (1)	30.6% (4)	20.3% (3)	30.3% (4)	33.0% (2)	32.0% (3)	31.7% (2)
Concerns about the person I provide care for	31.8% (2)	32.6% (2)	31.0% (3)	42.5% (2)	32.9% (3)	20.0% (4)	33.9% (1)	29.7% (4)	32.5% (2)	29.3% (3)
Information about financial support for medical expenses, from …	29.5% (3)	24.4% (4)	32.4% (2)	36.1% (4)	34.1% (2)	17.1% (8)	28.5% (5)	30.4% (3)	29.7% (5)	29.3% (3)
My own health problems	28.0% (4)	22.5% (6)	31.0% (3)	26.0% (8)	24.7% (7)	34.7% (1)	32.3% (2)	23.4% (6)	29.9% (4)	19.5% (5)
Lodging near hospital where the person I am caring for is treated	24.2% (5)	17.6% (12)	27.3% (5)	32.9% (5)	25.0% (6)	14.7% (11)	23.8% (8)	25.0% (5)	23.2% (8)	29.3% (3)
Feelings of vague anxiety	23.7% (6)	19.1% (8)	26.2% (6)	26.0% (8)	25.9% (5)	18.7% (6)	24.2% (7)	23.4% (6)	25.8% (6)	14.6% (8)
Help with my own relaxation and my personal life	22.6% (7)	18.7% (9)	24.7% (8)	32.4% (6)	23.5% (9)	11.8% (16)	25.0% (6)	20.4% (9)	23.9% (7)	17.1% (7)
Transportation service for getting to and from the hospital	21.6% (8)	24.2% (5)	20.3% (14)	27.4% (7)	21.2% (11)	14.9% (10)	22.0% (9)	20.7% (8)	21.5% (10)	22.5% (4)
Welfare services (e.g., psychological counselling) for caregivers	20.3% (9)	16.7% (14)	22.2% (10)	23.3% (13)	20.2% (12)	17.3% (7)	19.5% (12)	21.6% (7)	21.1% (12)	17.1% (7)
Feelings of anger, irritability or nervousness	20.0% (10)	16.9% (13)	22.2% (10)	24.7% (10)	21.2% (11)	13.5% (12)	20.3% (11)	19.8% (10)	21.8% (9)	12.2% (9)
A designated hospital staff member who would be able to provide…	19.5% (11)	13.3% (17)	23.6% (9)	21.9% (14)	23.8% (8)	12.0% (15)	22.0% (9)	17.1% (15)	19.9% (14)	18.0% (6)
Information about caregiving-related stress management	19.4% (12)	17.8% (11)	20.0% (15)	23.6% (12)	20.0% (13)	15.8% (9)	22.0% (9)	17.0% (16)	21.0% (13)	12.2% (9)
Information about tests and treatment	18.9% (13)	8.8% (26)	24.8% (7)	14.9% (24)	22.4% (10)	18.7% (6)	18.7% (13)	19.5% (12)	21.4% (11)	7.3% (12)
Depression	18.3% (14)	18.0% (10)	18.1% (17)	21.9% (14)	19.1% (14)	14.7% (11)	19.5% (12)	17.1% (15)	18.1% (17)	19.5% (5)
Loneliness or feelings of isolation	18.2% (15)	20.2% (7)	17.2% (18)	24.7% (10)	18.8% (15)	12.0% (15)	18.6% (14)	18.0% (13)	19.1% (15)	14.6% (8)
Information about the current status of the illness of the person I am…	18.1% (16)	11.1% (20)	22.1% (11)	15.1% (23)	21.2% (11)	17.3% (7)	20.3% (11)	16.1% (18)	19.9% (14)	10.0% (10)
Someone to help me with housekeeping and/or child care	17.2% (17)	17.6% (12)	16.6% (19)	27.4% (7)	5.9% (26)	19.7% (5)	21.0% (10)	12.5% (25)	16.8% (19)	19.5% (5)
Information about caring for the person with cancer (symptom….	17.2% (18)	9.9% (24)	21.2% (12)	20.3% (17)	18.8% (15)	13.2% (14)	17.7% (15)	16.8% (17)	18.8% (16)	9.8% (11)
Need for space reserved for caregivers	17.0% (19)	14.3% (16)	18.9% (16)	12.5% (28)	22.4% (10)	13.3% (13)	16.3% (16)	17.1% (15)	16.5% (20)	19.5% (5)
Seeing doctor quickly and easily when in need	16.4% (20)	9.9% (24)	20.7% (13)	18.9% (20)	19.1% (14)	11.8% (16)	13.7% (21)	19.6% (11)	17.9% (18)	9.8% (11)
Information about hospitals or clinics and physicians who treat cancer	15.6% (21)	12.2% (19)	17.2% (18)	20.8% (15)	15.3% (17)	10.5% (18)	13.8% (20)	17.9% (14)	15.4% (22)	17.1% (7)
Help with difficulties in family relationships after cancer diagnosis	15.5% (22)	14.3% (16)	15.1% (24)	25.7% (9)	14.1% (18)	7.9% (22)	16.1% (17)	15.0% (20)	14.7% (25)	19.5% (5)
Guidance about hospital facilities and services	15.3% (23)	11.1% (20)	18.1% (17)	19.4% (18)	18.8% (15)	8.0% (21)	16.3% (16)	14.4% (21)	15.0% (24)	17.1% (7)
Help with difficulties in interpersonal relationships after cancer…	15.1% (24)	16.5% (15)	13.7% (26)	24.3% (11)	12.9% (19)	9.2% (20)	14.5% (19)	15.9% (19)	15.2% (23)	14.6% (8)
Cooperation and communication among health care staff	14.2% (25)	11.0% (21)	16.4% (20)	16.2% (22)	17.7% (16)	7.9% (22)	12.1% (24)	16.8% (17)	14.7% (25)	12.2% (9)

^1^ Unmet need = items with a low, moderate or high need; … = item truncated; Shading represents items where 25% or more of each group reported this need.

**Table 5:** Frequency of unmet needs **^1^** according to patient disease characteristics.

	Overall Sample	Time since Patient Diagnosis			Cancer Type of Patient					
CNAT-C Item (25 Most Frequent Overall)	% (Rank)	0–6 Months (*n* = 85) % (Rank)	6–12 Months (*n* = 58) % (Rank)	>12 Months (*n* = 92) % (Rank)	Breast (*n* = 44) % (Rank)	Skin (*n* = 34) % (Rank)	Head & Neck (*n* = 25) % (Rank)	Prostate (*n* = 28) % (Rank)	Gynae (*n* = 21) % (Rank)	Other (*n* = 77) % (Rank)
Treatment near home	36.8% (1)	33.3% (1)	32.8% (2)	40.7% (1)	28.6% (3)	45.5% (1)	40.0% (1)	46.2% (1)	28.6% (2)	33.8% (3)
Help with economic burden caused by cancer	31.8% (2)	30.1% (2)	36.8% (1)	31.5% (3)	36.4% (1)	33.3% (2)	36.0% (2)	10.7% (11)	28.6% (2)	37.3% (1)
Concerns about the person I provide care for	31.8% (2)	29.8% (3)	31.6% (3)	33.0% (2)	25.6% (4)	29.4% (4)	40.0% (1)	25.0% (4)	25.0% (3)	35.5% (2)
Information about financial support for medical expenses …	29.5% (3)	26.2% (4)	29.8% (4)	30.4% (4)	29.6% (2)	30.3% (3)	36.0% (2)	21.4% (5)	19.1% (5)	30.3% (5)
My own health problems	28.0% (4)	25.0% (5)	21.1% (9)	33.0% (2)	18.6% (8)	17.7% (12)	28.0% (4)	35.7% (2)	35.0% (1)	31.6% (4)
Lodging near hospital where the person I am caring for is treated	24.2% (5)	21.7% (7)	24.1% (7)	26.4% (6)	20.5% (7)	29.0% (5)	28.0% (4)	28.6% (3)	19.1% (5)	20.8% (12)
Feelings of vague anxiety	23.7% (6)	23.8% (6)	22.8% (8)	24.2% (7)	18.6% (8)	17.7% (12)	24.0% (5)	21.4% (5)	15.0% (6)	31.6% (4)
Help with my own relaxation and my personal life	22.6% (7)	15.3% (15)	25.9% (5)	27.2% (5)	15.9% (11)	17.7% (12)	16.0% (8)	21.4% (5)	14.3% (7)	29.9% (6)
Transportation service for getting to and from the hospital	21.6% (8)	19.5% (8)	17.2% (14)	27.2% (5)	20.5% (7)	28.1% (6)	24.0% (5)	14.8% (8)	28.6% (2)	19.5% (13)
Welfare services (e.g., psychological counselling) for caregivers	20.3% (9)	16.9% (13)	17.5% (13)	23.9% (8)	16.3% (10)	28.1% (6)	24.0% (5)	10.7% (11)	23.8% (4)	18.2% (15)
Feelings of anger, irritability or nervousness	20.0% (10)	19.3% (9)	19.3% (11)	20.9% (12)	16.3% (10)	11.8% (16)	29.2% (3)	14.3% (9)	10.0% (8)	25.0% (7)
A designated hospital staff member who would be able to provide….	19.5% (11)	18.1% (11)	17.2% (14)	19.8% (13)	13.6% (13)	21.2% (9)	16.7% (7)	18.5% (6)	14.3% (7)	22.1% (11)
Information about caregiving-related stress management	19.4% (12)	17.9% (12)	19.3% (11)	19.6% (14)	20.5% (7)	12.1% (15)	16.0% (8)	10.7% (11)	14.3% (7)	25.0% (7)
Information about tests and treatment	18.9% (13)	19.1% (10)	17.2% (14)	16.3% (17)	2.3% (18)	26.5% (7)	12.0% (9)	21.4% (5)	9.5% (9)	23.7% (8)
Depression	18.3% (14)	13.3% (18)	19.3% (11)	22.0% (11)	18.6% (8)	17.7% (12)	28.0% (4)	11.1% (10)	15.0% (6)	18.4% (14)
Loneliness or feelings of isolation	18.2% (15)	10.7% (21)	21.1% (9)	23.1% (9)	20.9% (6)	11.8% (16)	28.0% (4)	3.6% (13)	15.0% (6)	22.4% (10)
Information about the current status of the illness of the person I am caring for …	18.1% (16)	16.7% (14)	14.0% (18)	18.5% (15)	4.6% (17)	20.6% (10)	16.0% (8)	18.5% (6)	9.5% (9)	22.4% (10)
Someone to help me with housekeeping and/or child care	17.2% (17)	9.5% (24)	20.7% (10)	22.8% (10)	22.7% (5)	15.2% (13)	16.0% (8)	14.3% (9)	14.3% (7)	16.9% (17)
Information about caring for the person with cancer (symptom management, …	17.2% (18)	15.3% (15)	20.7% (10)	15.2% (19)	4.6% (17)	20.6% (10)	16.0% (8)	21.4% (5)	9.5% (9)	20.8% (12)
Need for space reserved for caregivers	17.0% (19)	16.9% (13)	10.3% (21)	19.8% (13)	11.4% (14)	12.1% (15)	20.0% (6)	0.0% (14)	23.8% (4)	23.7% (8)
Seeing doctor quickly and easily when in need	16.4% (20)	10.6% (22)	24.6% (6)	16.3% (17)	9.1% (15)	20.6% (10)	8.0% (11)	10.7% (11)	10.0% (8)	23.4% (9)
Information about hospitals or clinics and physicians who treat cancer	15.6% (21)	11.9% (20)	14.0% (18)	17.4% (16)	15.9% (11)	21.2% (9)	8.0% (11)	14.3% (9)	19.1% (5)	13.2% (20)
Help with difficulties in family relationships after cancer diagnosis	15.5% (22)	10.6% (22)	20.7% (10)	15.2% (19)	15.9% (11)	5.9% (20)	20.0% (6)	17.9% (7)	4.8% (11)	18.2% (15)
Guidance about hospital facilities and services	15.3% (23)	14.5% (16)	14.0% (18)	15.2% (19)	9.1% (15)	18.8% (11)	16.0% (8)	14.3% (9)	9.5% (9)	18.4% (14)
Help with difficulties in interpersonal relationships after cancer diagnosis	15.1% (24)	12.9% (19)	17.2% (14)	15.2% (19)	18.2% (9)	5.9% (20)	24.0% (5)	7.1% (12)	0.0% (12)	19.5% (13)
Cooperation and communication among health care staff	14.2% (25)	8.2% (25)	19.0% (12)	14.1% (21)	18.2% (9)	14.7% (14)	8.0% (11)	10.7% (11)	4.8% (11)	14.3% (19)

**^1^** Unmet need = items with a low, moderate or high need. Abbreviation: Gynae = Gynaecological; … = item truncated.Shading represents items where 25% or more of each group reported this need.

## Data Availability

Data that support the findings of this study are available from the corresponding author upon reasonable request.

## Appendix A

**Table A1:** Factor loadings for confirmatory factor analysis model 1 and 2.

CNAT-C Item	Model 1	Model 2
Health and psychological problems		
My own health problems	0.653	0.656
Concerns about the person I provide care for	0.876	0.876
Depression	0.883	0.882
Feelings of anger, irritability or nervousness	0.947	0.946
Loneliness or feelings of isolation	0.925	0.925
Feelings of vague anxiety	0.933	0.934
Family/social support		
Help with over-dependence from the person I am caring for	0.878	0.878
Help with lack of appreciation of my caregiving from the person I care for	0.852	0.850
Help with difficulties in family relationships after cancer diagnosis	0.802	0.803
Help with difficulties in interpersonal relationships after cancer diagnosis	0.874	0.876
Help with my own relaxation and my personal life	0.964	0.963
Health-care staff		
Being respected and treated as a person by my doctor	0.940	0.940
Doctor to be clear, specific and honest in his/her explanation	0.839	0.840
Seeing doctor quickly and easily when in need	0.880	0.880
Being involved in the decision-making process in choosing any tests or treatments…	0.888	0.883
Cooperation and communication among health care staff	0.860	0.857
Sincere interest and empathy from the nurses looking after the person I am caring for	0.889	0.891
Nurses to explain treatment or care that is being given to the person I am caring for	0.872	0.873
Nurses to promptly attend to the discomfort and pain of the person I am caring for	0.794	0.796
Information		
Information about the current status of the illness of the person I am caring for…	0.968	0.969
Information about tests and treatment	0.950	0.951
Information about caring for the person with cancer (symptom management, diet, …	0.858	0.859
Guidelines or information about complementary and alternative medicine	0.736	0.735
Information about hospitals or clinics and physicians who treat cancer	0.739	0.742
Information about financial support for medical expenses, from government and/or …	0.853	0.853
Help with communication with the person I am caring for and/or friends and family …	0.805	0.779
Information about caregiving-related stress management	0.934	0.931
Religious/spiritual support		
Religious support	0.124	-
Help in finding the meaning of my situation and coming to terms with it	3.694	-
Hospital facilities and services		
A designated hospital staff member who would be able to provide counselling for …	0.891	0.888
Guidance about hospital facilities and services	0.918	0.913
Need for space reserved for caregivers	0.810	0.813
A visiting nurse service for the home of the person I am caring for	0.820	0.824
Opportunity to share experiences or information with other caregivers	0.767	0.770
Welfare services (e.g., psychological counselling) for caregivers	0.880	0.884
Practical support		
Transportation service for getting to and from the hospital	0.912	0.913
Treatment near home	0.796	0.795
Lodging near hospital where the person I am caring for is treated	0.743	0.743
Help with economic burden caused by cancer	0.905	0.901
Someone to help me with housekeeping and/or childcare	0.828	0.831
Assisted care in hospital or at the home of the person I am caring for	0.920	0.922

… = item truncated.

**Table A2:** Confirmatory factor analysis fit statistics.

	Model 1	Model 2
Chi square	155.88 *	1090.819 *
RMSEA (95% CI)	0.066 * (0.061–0.071)	0.050 (0.044–0.055)
CFI/TLI	0.915/0.908	0.955/0.951

* *p* < 0.01.
